# Editorial for “Materials Chemistry” Sections on *Molecules*

**DOI:** 10.3390/molecules25245833

**Published:** 2020-12-10

**Authors:** Assunta Marrocchi

**Affiliations:** Department of Chemistry, Biology and Biotechnology, University of Perugia, via Elce di Sotto 8, 06123 Perugia, Italy; assunta.marrocchi@unipg.it

Materials chemistry has been one of the most talked-about areas of materials research over the past decades. Spanning from polymers and composites to metallic, ceramic, and hybrid materials, as well as nano- and micromaterials, chemistry is unique in creating diverse arrays of new materials for applications in catalysis, energy conversion and storage, advanced electronics, environmental devices, drug delivery, smart textiles, packaging systems, or scaffolding for tissue engineering—to name but a few, with potential broad scientific and societal impact.

At the same time, matching materials and sustainability is becoming crucial, as a result of ever more stringent regulatory requirements in the European Union, North America and developed Asian countries [1,2,3,4]. These regulations have driven the development of new, eco-efficient and safe materials and technologies, following the principles of green chemistry [5] and green chemical engineering [6], to achieve enhanced functionality and environmental benefits.

Special importance is being attributed to the efficient use of the starting materials for obtaining the desired functional final products, while avoiding waste and the use of toxic substances, minimising the need for energy and using renewable resources [7,8,9,10]. As a potentially more sustainable alternative to conventional materials, for example, several bio-based materials and products have been introduced in the past few years in many areas, from agriculture to electronics, clothing or packaging [9,11,12,13,14]. Many sustainability-related education programmes have been introduced in undergraduate curricula to meet the ever-growing demand for green jobs in a large number of sectors, including the materials sector [15]. 

A selection of cutting-edge “Hot Papers” in the field of renewable materials, published in the Materials Chemistry Sector of *Molecules*, is reported in the Reference section of this Editorial [16,17,18,19,20,21,22,23,24,25].

The target of the “Materials Chemistry” Section in *Molecules* is to provide an open-access publishing platform for the effective dissemination of high-quality scientific outputs at the core of research on Materials Chemistry. The Section invites papers related to either experimental or theoretical studies about synthesis, properties, characterization, and application of materials in the widest sense (i.e., organics and inorganics, including—but not limited to—nanomaterials, hybrids, biomaterials, self-assembling systems of biologic and synthetic nature, thin-films).

What are the benefits of publishing in the “Materials Chemistry” Section of *Molecules*? Discoverability, to begin with. Open access, indeed, translates into wider readership, greater visibility and increased citations; moreover, the Editorial Team of Molecules ensures online dissemination through social media tools, to maximize the visibility of the published research. Next, the Journal is associated with a favorable and constantly increasing impact factor (3.267). Furthermore, the committment of the Journal to the peer-review process ensures the high quality of the article accepted and a very efficient timeline.

Materials Chemistry is experiencing a paradigm shift from traditional production technologies and practices to one that assigns value to the replacement of fossil fuels with renewable resources, waste minimization, and avoiding the use of substances that pose serious risk to human health and the environment. In this context, the prospect for research on alternative feedstocks, environmentally benign reagents, solvents, and catalysts, and safer and more readily recyclable products, as well as non-persistent, non-bioaccumulative, eco-compatible materials, looks exciting. We hope you will consider submitting your materials’ chemistry-related manuscripts to *Molecules*. Please also note that, in doing this, the “Materials Chemistry” Section should be selected from the drop-down menu.
